# Information synergy maximizes the growth rate of heterogeneous groups

**DOI:** 10.1093/pnasnexus/pgae072

**Published:** 2024-02-12

**Authors:** Jordan T Kemp, Adam G Kline, Luís M A Bettencourt

**Affiliations:** Department of Physics, University of Chicago, 5720 S Ellis Ave #201, Chicago, IL 60637, USA; Department of Physics, University of Chicago, 5720 S Ellis Ave #201, Chicago, IL 60637, USA; Department of Ecology & Evolution, University of Chicago, 1101 E 57th St, Chicago, IL 60637, USA; Mansueto Institute for Urban Innovation, University of Chicago, 1155 E 60th Street, Chicago, IL 60637, USA

## Abstract

Collective action and group formation are fundamental behaviors among both organisms cooperating to maximize their fitness and people forming socioeconomic organizations. Researchers have extensively explored social interaction structures via game theory and homophilic linkages, such as kin selection and scalar stress, to understand emergent cooperation in complex systems. However, we still lack a general theory capable of predicting how agents benefit from heterogeneous preferences, joint information, or skill complementarities in statistical environments. Here, we derive general statistical dynamics for the origin of cooperation based on the management of resources and pooled information. Specifically, we show how groups that optimally combine complementary agent knowledge about resources in statistical environments maximize their growth rate. We show that these advantages are quantified by the information synergy embedded in the conditional probability of environmental states given agents’ signals, such that groups with a greater diversity of signals maximize their collective information. It follows that, when constraints are placed on group formation, agents must intelligently select with whom they cooperate to maximize the synergy available to their own signal. Our results show how the general properties of information underlie the optimal collective formation and dynamics of groups of heterogeneous agents across social and biological phenomena.

**Significance Statement**

Current approaches to studying growth dynamics lack fundamental theory to explain the emergence of coordinated decision-making in groups of heterogeneous agents of arbitrary size. This work proposes a mechanism of information pooling, where the benefits of cooperation are described in terms of information synergy across agents’ collective signals in a complex environment. We show that more synergy, defined as information complementarity vs. a goal, results in the faster average growth of group resources. This introduces a principle of maximum synergy, which we show can be attained by learning over time and drives selective group formation. This work creates new insights into how structured organizations are created, and how they can be optimized over time in response to dynamical environments.

## Introduction

Collective behavior is a general feature of biological and social systems. It mediates the survival and evolution of populations under resource constraints, competition, or predation in natural systems (1) and the formation and persistence of social organizations in human societies (2). Much past work has modeled collective dynamics using homogeneous interaction rules, common to all agents, which are often phenomenological. While these models have produced diverse insights, they typically lack a theoretical foundation to explain how specific social behavior emerges among individual agents with heterogeneous information and behavior. Thus, significant knowledge gaps remain in most realistic situations, where agents with distinct but potentially complementary traits act collectively to maximize their joint growth (fitness, wealth) in knowable but stochastic environments.

Some examples help to illustrate the present situation. Game theorists and ecologists have considered many different cooperative interaction schemes (3) and explored evolutionary stable behavior (4), particularly on networks (5–7), where optimal behavior is identifiable under given interaction rules. Elaborating these schemes by introducing higher order interactions has broadened our understanding of more complex social networks (8–11), and their dynamical phase stability under varying interaction strengths (12). Researchers have also studied, both theoretically and in the laboratory, how memory of previous interactions influences agents’ preferences for future encounters (13–16), the spread of social crises across distance (17), and the formation and scaling properties of social collectives (18, 19), such as cities (20, 21).

In addition to interaction rules and associated payoffs, collective dynamics is predicated on maximum principles, which specify agents’ preferences in view of a goal and thus render their behavior intelligent (optimal). For example, inclusive fitness theory, which assumes a reproductive benefit to cooperation because of shared genes (22, 23) has been studied in mixing populations and over networks (24) where it predicts population benefits to cooperation through several forms of reciprocity (25). More recently, researchers have studied resource pooling in models of growth as a means to minimize environmental uncertainty and associated loss of fitness among agents experiencing independent fluctuations with shared statistics (26, 27). Such approaches remain limited by the association between collective behavior and (genetic) homophily. Still, they can help to explain the existence of phase transitions in cooperation networks (12, 19), and specify agents’ plausible behavioral patterns (15), even if doubts remain about inclusive fitness’s predictive power (28).

Generally, however, most current quantitative frameworks fail to address collective dynamics when agents remain heterogeneous across skills, knowledge, and behavior (29–31). Developing more general approaches to collective behavior that include adaptation along with heterogeneity is a crucial step toward understanding how agents self-organize in more complex and dynamical environments, where specialization and the division of labor and knowledge become key.

Adaptive behavior requires agents to acquire and process information over time (32, 33) in response to their environments and each other. In realistic situations, limited experience, specialization costs, and physical limitations of effort, energy, and time, all prevent agents from perfecting their knowledge of complex environments (34, 35). A natural way to mitigate these individual limitations is to pool knowledge across agents leading to the formation of social organizations (36), and the division and coordination of labor in terms of their behavior (37). This is widely observed in human organizations and animal social behavior starting with the division of labor by age and sex.

By working jointly to predict characteristics of their environment (31) and gather resources, groups of agents can maximize their collective fitness even when each individual has very limited knowledge. In a setting where there are resource returns to successful prediction and behavior, information of the state of a statistical environment determines the fitness of the population (38, 39), though there are questions about how such benefits emerge quantitatively (40). Here, we formalize the calculation of these social benefits in terms of the properties of information and show how maximizing knowledge complementarities (synergy) maximizes the long-term growth rate of collectives. Specifically, we derive an expression for the additional payoff to cooperative behavior in terms of the joint information synergy about the agents’ dynamical environment.

The aggregation of dispersed, tacit information among a group of agents has long been proposed as the principal role of economic markets (41), operating through the price mechanism. In such settings, a public price forms as the result of the allocations of traders with diverse knowledge, buying and selling an asset according to their beliefs (estimates) of its value. Several types of markets, both centralized (42–44) and decentralized (45–47), have been discussed as efficient aggregators of information in this sense, but fundamental objections have also been raised (48). Information, in the sense of this *efficient markets hypothesis*, usually reflects only average beliefs among traders (49). In contrast, our approach shows how dispersed knowledge can be combined in optimally predictive ways.

These results lead us to introduce the principle of maximum synergy, which maps the maximization of pooled resource growth rates into optimal social interaction structures. This work adds new dimensions to the study of collective dynamics by connecting the structure of groups to that of information in complex environments mediated by agents’ diverse subjective characteristics, such as their present knowledge and information acquired as the result of diverse experiences throughout their life course.

## Theory of collective growth

We start by demonstrating how the benefits of collective action emerge from pooling information in synergistic situations. Synergy means the combination of behavior, knowledge, and skills that complement each other toward a goal. This concept is necessary for creating effective organizations that embody complex information (31), but it is often not sufficiently formalized in common language, such as in discussions of innovation (50) or firm structure.

Here, we will refer to synergy as an explicit information-theoretic quantity that measures the additional predictive power that a group acquires upon pooling its agents’ information, relative to the knowledge of each individual separately. This quantity has been introduced some time ago in the context of studying circuits in information processing systems (51, 52), and has provided a framework for studying higher order neuron interactions in the brain (53), and causality and information in complex systems (54, 55). As we will show, synergy results formally from the conditional dependence between the probability of predictive signals distributed in a population and events in a shared environment. The gain in predictive power from agents pooling information as collectives allows them to obtain additional resources from a knowable environment beyond what agents alone can do, thus boosting their fitness or productivity.

It follows that collectives that seek to maximize their resources over long times must combine the information from their agents’ individual models of the world in a way that accesses the most synergy. Groups that do not know a priori how to realize their synergies must discover how to do so, by adjusting their collective knowledge and interaction structure while observing outcomes of their environment in an iterative learning process. After developing the general framework for group formation and collective growth across group sizes, we demonstrate a model environment that exhibits synergy using logic gates. We will also demonstrate how synergy scales with the number of unique signals in a collective, and how specific combinations of signals affect the average growth of resources for the group.

### Collective growth in synergistic environments

We consider a population of *N* agents, each with initial resources $r_{i},\;i=1,\ldots ,N$ that can be (re)invested into the set of outcomes of their environment to generate returns. Each agent has access to a private signal (their knowledge), s∈S, which is used to predict the state of the environment and make resource allocations to possible outcomes e∈E. This signal may represent several different processes such as sensory input or a lead retrieved from memory. With optimal parameterization of a model of the environment, P(E∣S), an agent’s optimal investment strategy leads to an average resource growth rate (over time) γ=I(E;S) (38), where I(E;S) is the mutual information between environmental states *E* and the agent’s signals *S*. (We are working in units of units time t=1, for simplicity.) Agents with better models (and better statistical estimations of P(E∣S)) thus experience higher average growth rates.

We now define the agent’s environment more explicitly, by a set of *l* distinct signals with unique statistics, $S\equiv \{S_{1},\ldots ,S_{l}\}$ as P(E∣S), with marginals of events P(E) and signals P(S). The joint information that the universe of signal, S, has on *E* is at least equal to each of the signals $S_{j}$, that is $I(S;E)\geq I(S_{j};E)$, for all *j*. Generally, this inequality is strict if the conditional information I(S∣E)>I(S) (51, 52). We compute the total information by summing over the mutual information between each of the signals independently, subtracted by an interaction term across them,


(1) $$I(E;S)=\sum_{j}I(E;S_{j})-R_{P}.$$

The coefficient of redundancy, $R_{P}$, measures the strength of this conditional dependence across larger sets of signals (two, three, etc). It is defined in SI Appendix A


(2) $$\begin{array}{rl} R_{P} & =\sum_{\;j>k=1}^{l}R(E;S_{j};S_{k})+\sum_{\;j>k>m=1}^{l}R(E;S_{j};S_{k};S_{m})\\ & \;+\cdots +R(E;S_{1};\ldots ;S_{l}). \end{array}$$

The coefficient of redundancy can have a positive or negative value, indicating different conditional relationships between the signals and environmental states. When $R_{P}>0$, there is information between signals irrespective of environmental events. This means that signals are partially *redundant*, and consequently, there are diminished returns to pooling information as $I(E;S)<\sum_{j}I(E;S_{j})$. Conversely, when R(E;S)=0, the signals are statistically independent, and the benefits of pooling information increase linearly with the information of each signal on the environment but there is no synergy. Finally, when R(E;S)<0, there is conditional dependence of the signals on the environment. This is called *synergy* and yields a superlinear benefit to pooling information in the number of agents, above and beyond the information contributed from each signal individually.

#### Group formation and collective decision-making

We have now defined individual resource growth rates as a quantity of information and discussed how information can be aggregated across different signals to express their synergy relative to states of the environment. Now we can explore how agents with different signals can pool information together as coordinated groups, and access the synergy in their environment through collective decision-making.

Consider the undirected hypergraph H=(A,G) of vertices, *A*, and hyperedges *G*. We consider a discrete number of vertices, $A=\{a_{1},a_{2},\ldots ,a_{N}\}$, where $a_{i}$ identifies agent *i*. The set of hyperedges, g∈G={1,2,…}, called groups, defines the number of cooperating collectives. A hyperedge connects $1\leq N_{g}\leq N$ agents. We assume that agents can only belong to a single group. Therefore, by construction, $\sum_{g}N_{g}=N$ and the sum over all nodes of every hyperedge yields the number of agents in the population. There exist two extremes of cooperation. First, when a single hyperedge spans every node, meaning all agents pool information in a single group. In the limit of no cooperation, $N_{g}=1$ for all *g*, and no agents pool information. In this case, the dynamics of the model are similar to previous work (38).

Let $S_{g}$ be the set of unique signals held by the agents of a group *g* to be pooled, such that $S_{g}\subseteq S$. The number of *cooperants* is defined by the number of unique signals, $|S_{g}|=k_{g}$, and is bounded by $1\leq k_{g}\leq l$. When $k_{g}=l$ and the group has a complete signal, the collective can make maximally informed decisions. Conversely, when $k_{g}<l$, the signal is considered *incomplete*, and the collective can only interpret and act on a subset of signals. As we will see, the number of unique signals a collective can observe determines the amount of information they can access.

Now that we have defined how agents organize into groups of various sizes, we can discuss how agents pool their information to make collective decisions and grow their resources in dynamic environments. At every time step, a collective with access to all signal types observes a unique private signal $s=\{s_{1},\ldots ,s_{l}\}\in S$. Each agent then allocates its resources $r_{i}$ on events according to collective *g*’s allocation matrix B(E∣s). As the event *e* is observed, the agent is rewarded with returns $w_{e}$ to the fraction of resources invested in *e*, B(e∣s). In the limit of many sequential investments *n*, the average growth rate of resources converges to


(3) $$\gamma =\frac{1}{n}\log\;\frac{r_{n}}{r_{i}}\approx \sum_{e,s}P(e,s)\log\;[B(e|s)w_{e}].$$

The optimal investment in the large *n* limit is the conditional probability of the event given the signals, B(e∣s)=P(e∣s). When the rewards are “fair,” and $w_{e}=1/P(e)$, the optimal growth rate is given by the mutual information (56) defined in Eq. 1, γ=I(E;S).

The typical collective may not have a complete signal, and instead may only observe and interpret a subset of all unique signals $S_{g}$. Their optimal allocation, given by $P(E|S_{g})$, then has mutual information $I(E;S_{g})\leq I(E;S)$, with equality only if the omitted signals are completely redundant with present signals. Unless there are redundant signals, an incomplete group is guaranteed to have suboptimal information and growth rate.

**Fig. 1.**
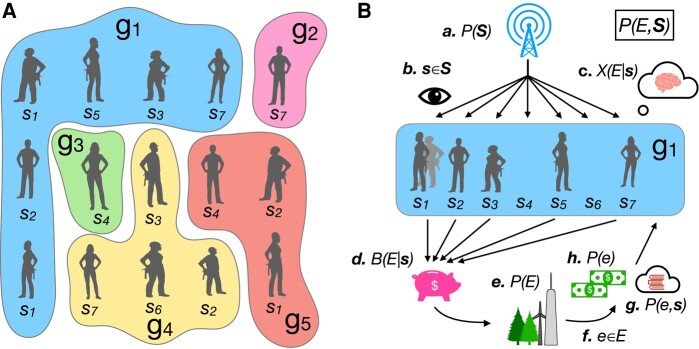
Groups of agents with different signals grow resources based on the information between their signals and states of the environment. A) Groups, denoted by *g*, are composed of an arbitrary number of agents. Each agent belongs to only one group and can observe and contribute one signal to the group. A group contains $k_{g}$ unique signals. B) At each time step, (a) the group’s private channel outputs a signal s∈S with probability P(s). (b) Each member of the group observes their signal $s_{j}$, and (c) the group consults their collective belief for the conditional outcome probability of the environment, X(E∣s). (d) The agents make proportional resource allocations on all possible outcomes B(E∣s). (f) and (e). The true event e∈E is observed in the environment with probability P(e), and (g) the agents receive payouts proportional to the marginal probability of *e*.

Agents a priori may also not have perfect knowledge and must invest using their best estimate of the true conditional probability, $X(E|S_{g})\neq P(E|S_{g})$. In this case, the collective’s average growth will be submaximal by the number of signals and lack of information on signals and is described by


(4) $$\gamma_{g}=I(E;S_{g})-{\text{E}}_{s_{g}}(D_{\mathrm{KL}}[P(E|s_{g})\|X(E|s_{g})]),$$

where $E_{s_{g}}$ is the expectation value over the states of the group’s signals, and $D_{\mathrm{KL}}[P(E;s_{g})\|X(E;s_{g})]=\sum_{e}P(e|s_{g})\log\;(P(e|s_{g})/X(e|s_{g}))\geq 0$ is the Kullback–Leibler divergence, an information measure expressing how similar the distributions are. This result shows that collectives with both a better model as reflected by the first term, a better characterization of the model and its various synergies by the second, and a more complete signal, will experience higher growth rates. Furthermore, $\gamma_{g}<\gamma$ unless *g* is the full set of signals, so it is typically valuable to add more signals to the group. This setup is illustrated in Fig. 1.

### Maximum synergy principle and optimal growth

These results introduce important considerations for how collective innovation and growth determine strategies for group formation. In theories of cooperation such as kin selection (57) and scalar stress (58), group formation is advantaged by member relatedness and disadvantaged by unfamiliarity. This is intuitive in many situations, as agents are more likely to cooperate when they are more certain others will reciprocate (59), and cooperating with similar agents may naturally minimize this uncertainty. Equation 4 counters this intuition by defining an explicit benefit to cooperating with dissimilar agents across heterogeneous, complementary skills, and information. Specifically, a group with more synergistic signals, as defined through the conditional dependence of their decisions on states of the environment, will experience higher growth. So, even if there are additional coordination costs for more heterogeneous agents, there is now a possibility that cooperation will emerge as there are also greater informational benefits, formalizing intuitive ideas about the value of diversity (60).

The beneficial contribution of synergy to the growth rate of resources provides an important input to models of random multiplicative growth, such as those commonly used to study wealth dynamics and mathematical finance. In its simplest form, the stochastic growth rate in such models is characterized by its first two temporal moments. The average over time, *η*, and the resource temporal SD (volatility), *σ*, combine under Itô integration to give the actual growth rate $\gamma =\eta -\sigma^{2}/2$. Maximizing this growth rate (as a positive quantity) entails maximizing *η* and minimizing *σ*, which at the individual agent level can be achieved by (Bayesian) learning over time (38).

At the population level, it has been proposed that pooling resources in groups would naturally emerge as a means to reduce *σ*, when growth rate fluctuations are independent across agents, and thus maximize *γ* (26, 61).

Our results introduce a different possibility of cooperation, through pooling information in structured groups, that maximizes *η* (and *γ*) through synergy effects. Thus, to maximize *γ*, agents should pool information with the most diverse set of collaborators possible to access the most mutual synergy, viz. the environment. This *maximum synergy principle* defines the benefit of intelligent collective behavior in complex environments where there are agent-level limitations to knowing the environment fully and where mechanisms of the division of labor and knowledge are favored. This principle is general and applies across levels of cooperation, whether it be individuals matching skills to form groups or specialized groups organizing into more complex collectives (40), all the way to large-scale societies.

**Fig. 2.**
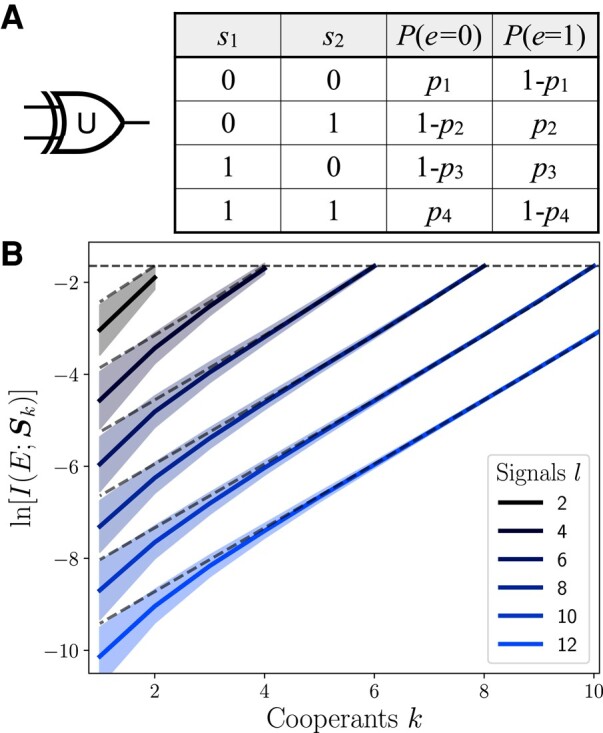
Complementary strategies for increasing the long-term growth rate of resources from the environment in stochastic growth models. Pooling resources can reduce volatility through a hedging strategy, while pooling information creates synergy to increase average growth rates. The lines represent contours of constant average growth rates *γ*.

Generally, these two strategies, information synergy vs. resource pooling under independence, are distinct modes of cooperation over which groups can maximize *γ*, as demonstrated in Fig. 2.

As we will see later, the decision of whom to cooperate with is not trivial, as different combinations of signals may yield varying synergies. This means that under constraints to group size such as from cooperation costs per connection, groups satisfying the maximum synergy principle must intelligently select which signals and agents to integrate, and which to exclude as redundant.

Furthermore, collectives may not a priori know the optimal allocation strategy that leverages the synergy available to their signals, meaning that intelligent collective behavior must itself be learned over time and by exploring the best possible matchings. We will now develop the dynamics of how a group maximizes its synergy given a set of signals.

#### Synergy maximization through Bayesian inference

Bayesian learning is the optimal strategy to incorporate new information from observed events into the estimate of conditional probabilities, such as those of environmental states given agents’ signals (38). Agents can also learn the synergy embedded in their environment in groups by collectively weighing their conditional observations across their individual signals. A group wanting to maximize their synergy must then update their conditional relationship through a Bayesian inference process


(5) $$X_{n}(e|s)=AP(s_{n}|e_{n})X(e_{n})=\left[\prod_{i=1}^{n}\frac{P(s_{i}|e_{i})}{P(s_{i})}\right]X(e),$$

where the normalization $A=(\int de_{n}P(s_{n}|e_{n})X(e_{n}){)}^{-1}$. We take the prior probability, $X(e_{1})=X(e)$, because we are assuming that the environment is stationary or at least slowly changing relative to groups’ learning rates.

Bayesian inference converges X(E∣S)→P(E∣S) over time, decreasing the information divergence, and maximizing synergy and average growth. For groups with incomplete signals, the information acquired through learning is still bounded by what is available in the incomplete signal space.

We have thus far defined collective growth in terms of information synergy, and shown how agents can learn as a collective to increase their growth rate over time. We will now illustrate these general results using a model based on logic circuits.

## Modeling synergy with logic circuits

Logic circuits have been used extensively as models for synergistic interactions (40, 51, 52). This is because their outputs are predicted by combinations of inputs, much like events are predicted by combinations of signals. Among other logic circuits (like AND or OR), the XOR gate is unique in that information between inputs and outputs only exists as synergy across all inputs (62); no individual input has mutual information with the output.

In the following, we will show how modifying the XOR gate relaxes this condition, such that information exists for any input and scales on average with the number of cooperating signals. Similar to Ref. (38), while this model will be used to study synergy in a simplified setting, the theory is defined for general dynamical environments.

### The uniform XOR gate

Consider the space of statistically independent binary signals $s_{j}\in 0,1$, such that a sample set s has uniform probability $P(s)=2^{-l}$. We assign each input s a binary event, e∈0,1, using the generalized XOR rule, $e=M_{2}(s)\equiv [\sum_{\;j=1}^{l}s_{j}](\mathrm{mod}\;2)$ with binomial probability $p_{s}$. From the sets of sampled signals, s, and binomial coefficients $p=\{p_{s}\}$, we can define this generalized XOR circuit as a joint distribution on signals and events as


(6) $$P(E,S|p)\equiv f(p,l)=\frac{1}{2^{l}}\prod_{s}(p_{s}{)}^{M_{2}(s)}(1-p_{s}{)}^{1-M_{2}(s)}.$$

**Fig. 3.**
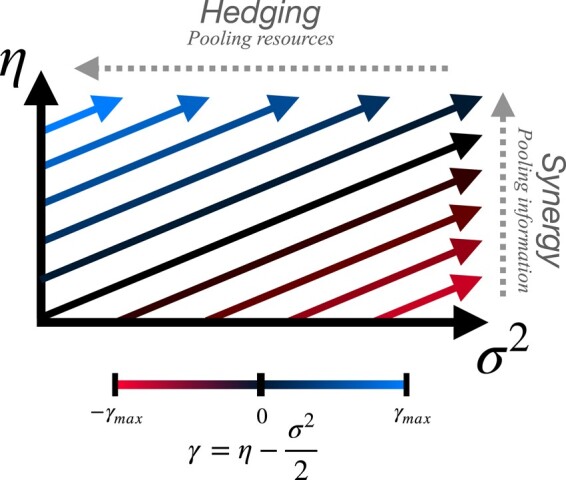
The UXOR model provides an environment for exploring synergy across groups of arbitrary size. A) The UXOR circuit, demonstrated by the modified XOR symbol, and its truth table for l=2. B) The information of a circuit of size *l* scales exponentially in cooperants, *k*.

This distribution is called the uniform XOR (UXOR). It performs a unique, *l* dimensional XOR gate on each input s with probability $p_{s}$. When $p_{s}=1$ for all input permutations, this circuit behaves deterministically like an XOR gate, and the complete group has 1 bit of information. In the limit of $p_{s}=0.5$, this no longer models a logic gate as the output is uncorrelated to the inputs. The truth table of this circuit is shown in Fig. 3A for an environment with two signals.

#### Information scaling in the UXOR environment

With this explicit choice of distribution, we can explore quantities of information that will define a group’s growth process. For simplicity, we choose a uniform prior for the distribution of p, but in principle any prior distribution is admissible. The information available in the environment measures the maximum average growth rate a group with a complete signal can experience. When averaged over all configurations of p, the information is given by I(E;S)=log2−1/2≈0.28 bits (SI Appendix C).

For groups with incomplete signals (when $k_{g}<l$), we compute the information by marginalizing Eq. 6 over the $\lambda_{g}=l-k_{g}$ signals unavailable to the group. The procedure for marginalization is defined in SI Appendix C, but in general, marginalization of one signal halves the size of the parameter space p that describes the distribution. The average information for an incomplete signal is approximately (SI Appendix D)


(7) $$I(E;S_{g}|p)\approx 2^{-\lambda_{g}}\left(\log\;2-\frac{1}{2}\right).$$

Average information scales exponentially, $∼2^{k}$, as more signals are included. The mutual information of the complete signal is independent of the number of signals, so the information of a single signal must converge to zero in the limit of large *l*.

The exponential scaling of the information with the number of cooperants is demonstrated in Fig. 3B, as lines on a logarithmic scale for environments of increasing *l*. The curves are computed by Monte Carlo sampling circuits for *l* signals by measuring the information after λ=l−k marginalizations.

#### Growth and group learning

Until now we have explored the mean behavior of this environment subject to a uniform prior. In general, collectives do not have perfect information on a single prior. In this case, their inaccurate guess for the set of binomial coefficients is parameterized by $x_{g}\equiv \{x_{s_{g}}\}$, indexed by the signals available to the group $s_{g}\in S_{g}$, and the collective’s likelihood model becomes $X(e|s_{g})=f(x_{g},k_{g})$. The information divergence term of Eq. 4 becomes the divergence between $f(x_{g},k_{g})$ and $f(p_{g},k_{g})$, where p has been projected into the subspace spanned by $S_{g}$, averaged over all signals ${\text{E}}_{s_{g}}[D_{\mathrm{KL}}]=\langle p_{s_{g}}\log\;(p_{s_{g}}/x_{s_{g}})+(1-p_{s_{g}})\log\;[(1-p_{s_{g}})/(1-x_{s_{g}})]\rangle$ here angle brackets denote sample averages over the binomial values. Subtracting the mutual information by this term yields the growth rate under imperfect, incomplete group information.


(8) $$\gamma_{g}=\left\langle p_{s_{g}}\log\;x_{s_{g}}+(1-p_{s_{g}})\log\;(1-x_{s_{g}})\right\rangle +\log\;2.$$

We have so far described growth rate dynamics under a stationary $x_{g}$. To illustrate growth dynamics under group learning, we turn to the Latent Dirichlet Allocation (LDA) model. Through a categorical description of pairs of events and signals, agents experience average dynamics to $x_{g}$ in the limit of high sampling rate ω=n/t≫1


(9) $$x_{g}(t)=\frac{p_{g}t/2\kappa +x_{g}}{1+t/2\kappa },$$

where *κ* defines the Bayesian update time. The details of LDA are given in Ref. (38) and provide parametric dynamics that converge to full information as a power law in time, in stationary environments.

To study resource dynamics in the UXOR environment, we simulated agent investments in a Monte Carlo sampled environment. We randomly assigned N=5,000 agent signals in an l=4 environment, then randomly assigned them to groups sized $l\leq N_{g}\leq 11$. This results in an ensemble of groups with cooperants $1\leq k_{g}\leq 4$. We reveal Bernoulli-sampled signals to the groups, whose agents collectively decide on which events to allocate resources to. For each group, we track the resources of a representative agent, informed by the group, investing their individual resources through time. The full details of the setup are provided in the Supplementary material.

**Fig. 4.**
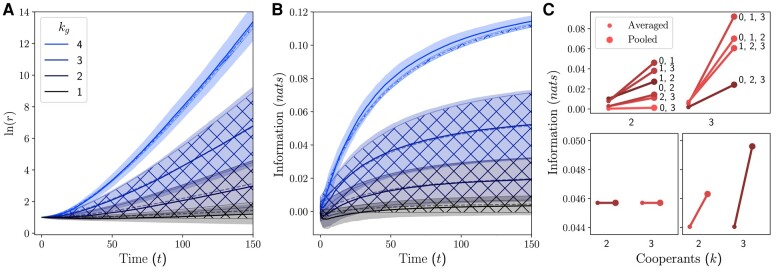
Groups learning an l=4 environment using more unique signals acquire more resources and information, but combinations of signals have unique amounts of information. A) Temporal resource trajectories, grouped by number of unique signals in the corresponding group show that growth increases with the number of signals. B) Groups with more signals can gather more information from the environment. There is high variability when $k_{g}<l$, as different combinations of signals access different amounts of information. C) Top For $k_{g}=2,3$, the synergy benefits of a parameter configuration are given by the difference between the information when averaged (small dot) and pooled (large dot). Bottom Parameter values exist where no signal combinations hold synergy (left) and synergy is equivalent across signal combinations (right).

Figure 4 illustrates the results of this simulation. In panels A and B, the Monte Carlo simulated means are shown as solid lines, with 95% CI shaded regions. Theoretical means are computed from the initial population configuration using Eq. 9, plotted as dashed lines, with hash-filled uncertainty regions. Simulated groups have randomly assigned members with uniformly assigned signals, where N=2,000. The more unique signals a group can access, the more they can learn, and the more resources they acquire over time. A high signal-to-noise ratio when $k_{g}=1,2$ causes growth rates to be lower than the theoretical mean, and cumulatively results in fewer resources over time.

#### Constrained intelligent group formation

For the groups with $k_{g}<4$ (incomplete signals), there is a significantly higher variance in both information and resources compared to $k_{g}=4$. This is attributed to differences in synergy between groups with different combinations of signals of order *k*. This illustrates a general feature of the maximal synergy principle; that signal combinations with higher conditional dependence on the environment will have higher synergy and experience higher growth rates than other combinations. Figure 4 demonstrates the synergy effects across different combinations of signals. For each group of size *k*, the left, smaller dot indicates the amount of information each signal has averaged over the signals present. The right, larger dot indicates the total information the combination of signals has when pooled. The difference between the two dots gives the amount of synergy. We see, for example, that even though signals 0 and 3 have less information than signal 2, both signals have higher synergy effects when pooled with 1 individually, as indicated by their crossover with the 1, 2 line. For a group aggregator, not only does this mean that signal choice is nontrivial but also that individual information is not generally a good indicator of synergy benefits that can be realized when pooled.

As demonstrated by the bottom plots in Fig. 4C, through a suitable selection of p, we can also design special environments such as where either no synergy is present, or where there are uniform benefits of synergy across combinations of signals. The procedure for constructing environments with specific synergy profiles will be developed in future work.

These results point to the challenges of leveraging the full complementarity of available signals in practice, toward satisfying the maximum synergy principle in organizations. For example, novel signal identification may result in disruptions of existing organizational structures, which while ultimately optimal may not be realizable without some sacrifice of short-term efficiency or increased costs. Maximizing long-term synergy and growth entails a tradeoff, since over shorter horizons exploitation of existing knowledge may be preferred both individually (63) and as organizations adapt structurally to the specialties of its members (64), which may vary depending on the complexity of the environment (65). We have also shown that organizations that match the complexity of their environment through appropriate personnel specialization and integration (minimizing the $D_{\mathrm{KL}}$ with the environment) experience the fastest growth, in agreement with the analysis of empirical data (66).

## Discussion

In this paper, we developed a novel mechanism of cooperation among heterogeneous agents that use shared information to grow resources in stochastic but knowable environments. We derived the benefits of cooperation in terms of synergy gained by pooling information across agents’ unique signals and its consequences for the growth rate of collectives. This motivates the principle of maximum synergy, whereby a group’s aggregate growth is highest when it maximizes the synergy of its members relative to a statistical environment. We proposed this principle as a complementary avenue to cooperation resulting from the reduction of volatility through resource pooling in multiplicative growth models. We then showed that a group with no a priori knowledge of its potential synergy can learn it through Bayesian inference. We illustrated these principles using a model of a high-dimensional probabilistic logic gate and showed that, on average, group synergy scales superlinearly with the number of unique signals in the group. We also illustrated the challenge faced by groups incurring size-related costs to pick not just unique signals but also admit new group members as additional signals that maximize their potential collective synergy.

These results formalize several insights into the causes and benefits of cooperation. First, the formal properties of information allow us to consider how the limits to human effort and ability motivate group formation. Specialization through learning or adaptation is costly in terms of time and resources, motivating a division of labor to fully learn and maximize productivity across disparate but synergistic agents (36). This motivates the formation of heterogeneous cooperation networks (67, 68), where agents seek new connections that complement their particular signals and that vary conditionally on local environments. In this sense, collectively navigating complex fitness landscapes is naturally achieved by satisfying the maximum synergy principle. However, maximizing synergy can be a challenging and costly task for groups because it requires time, effort, and social rearrangement to learn the complementarities among a set of signals.

Second, these results motivate analyses of how information and resource pooling strategies affect different levels of selection within an organizational hierarchy. Effective resource pooling relies on uncorrelated fluctuations across participants, which is not possible when agents are making coordinated decisions across signals. We therefore expect information and resource pooling strategies to create tradeoffs in group formation, and apply to different environmental features and levels of selection.

Groups lacking informational complementarities (because they are homogeneous) operating in very variable environments should pool resources to minimize volatility. This may apply to people in insurance pools, or independent economic sectors within a common population, such as a city or nation. Conversely, groups in complex environments made up of agents with complementary knowledge, such as within a firm or innovation ecology, should engage in information pooling and skills specialization to maximize their collective production potential whenever the variability of the environment and costs of cooperation are sufficiently low.

Parsing out these modes of cooperation becomes more important when considering how groups respond to changing environmental or social conditions. As new environmental conditions emerge, such as new industries or technologies, the distribution of synergy across different group configurations will also change, selecting for different group compositions and skill combinations. This has the interesting implication that new knowledge (science, technology, institutional change) should be disruptive of established social and economic structures explicitly because it enables new synergies and faster growth. This also has implications for natural ecosystems (69) where changing environmental conditions, such as via climate change, and adaptation may alter the relative fitness of their components and thereby their overall structure.

Third, the framework developed here describes a general approach to interaction dynamics in many fields. The conditional probabilities P(e∣s) capture the general structure of information between populations’ signals and actions, and their environment. Through synergy maximization, that information becomes encoded in how groups form and are structured, and which sets of coordinated behaviors produce beneficial or detrimental outcomes across agents. By averaging over environments, we can produce a set of rules for (average) rewards associated with agents’ perceptions and actions. This shows how general conditional probabilities of choices and behaviors in given environments may underlie particular “games” and other phenomenological agent interaction rules (70).

In this sense, several interesting themes in the collective dynamics of iterated games may be relatable to conditional probabilities and growth rates set by information. Two aspects of this general problem that we did not discuss here are the distribution of payoffs from collaborative action back to individual agents, and the (short-term) advantages of defection. The emergence of trust (71–74) among agents necessary for realizing long-term higher growth rates is likely costly and may benefit from an aggregator that can reduce the associated risk. This catalyst of long-term synergy can also be applied to models of interaction among risky innovators (75), where coordinators can actively influence selection by managing interfirm links and information access. In environments with conditionally dependent signals, agents may also learn to predict other agents’ behavior leading to the emergence of local trust clusters (76) without the presence of an aggregator.

Thus, although the principle of maximum synergy is general, there are multiple obstacles to realizing it in practice. Pathways to explore latent synergies must overcome short-term costs of learning and discovery, coordination, social inclusion, and exclusion, and promote the long-term bonds necessary to derive collective benefits, which once created must also be distributed fairly. When the balance of these benefits and costs is positive and can scale up, synergy becomes naturally expressed in higher order interactions as is observed in generalized reciprocal cooperation and the emergence of complex cultures as interdependent knowledge and behavior among many agents (25).

In summary, the formal properties of information, made explicit over group structures and time, provide the theoretical basis for a broad class of agent interaction models found throughout the social and ecological sciences. This includes the formation of complex societies made up of diverse cooperating agents in situations where large-scale synergy becomes possible and can be maximized.

## Supplementary Material

pgae072_Supplementary_Data

## Data Availability

The data underlying this article are available with DOI/accession number: 10.5281/zenodo.8347077.

## Preprints

This manuscript was posted on a preprint: https://doi.org/10.48550/arXiv.2307.01380.
