# Supplementary material for: Information synergy maximizes the growth rate of heterogeneous groups
Source: PNAS Nexus. 2024 Feb 12;3(2):pgae072. doi: 10.1093/pnasnexus/pgae072 (PMC10901557; doi:10.1093/pnasnexus/pgae072)
Supplement: pgae072_Supplementary_Data [file pgae072_supplementary_data.zip › PNASNEXUS-PNASNEXUS-2023-00975R-s01.pdf]

# Appendix for: Information Synergy Maximizes the Growth Rate of Heterogeneous Groups

Jordan T. Kemp[1], Adam G. Kline[1], and Luís M. A. Bettencourt[2,3]
[1] *Department of Physics, University of Chicago, Chicago, Illinois 60637, USA*
[2] *Department of Ecology and Evolution, University of Chicago, Chicago, Illinois 60637, USA and*
[3] *Mansueto Institute for Urban Innovation, University of Chicago, Chicago, Illinois 60637, USA*
(Dated: February 7, 2024)

## I. APPENDIX

### A. Information Aggregation

Consider a target statistical variable $E$ (environment), that we wish to predict using $l$ other variables (signals) $\boldsymbol{S} = \{S_1, \dots, S_l\}$. The mutual information between each signal $S_i$ separately and $E$ is given by [1]

$$I(E; S_i) = H(E) - H(E|S_i) = -\frac{\Delta H(E)}{\Delta S_i}. \tag{1}$$

where $H(E)$ is the Shannon entropy of $E$, and the variation measures the difference in entropy of the event when conditioned on the signal. From the rules of information aggregation, this expression generalizes to information across every added signal [2]. The mutual information between the event and the set of several signals is given by

$$I(E; \boldsymbol{S}) = -\sum_{i=1}^{l} \frac{\Delta H(E)}{\Delta S_i} - \sum_{i>j=1}^{l} \frac{\Delta^2 H(E)}{\Delta S_i \Delta S_j} - \dots - \frac{\Delta^l H(E)}{\Delta S_1 \dots \Delta S_l}. \tag{2}$$

The first term of this expansion is just a sum over the mutual information of each individual signal and the environment. The goal of this section is to show that the inclusion of each new signal introduces a coefficient of redundancy of progressively higher order. The first term is

$$\begin{aligned}\frac{\Delta^2 H(E)}{\Delta S_i \Delta S_j} =& H(S_i) - H(S_i|E) + H(S_j) - H(S_j|E) - H(S_i, S_j) + H(S_i, S_j|E) \\ =& H(S_i) - H(S_i|E) - H(S_i|S_2) + H(S_i|S_j, E) \\ =& I(S_i; S_j) - I(S_i; S_j|E) \equiv R(E; S_i; S_j),\end{aligned} \tag{3}$$

where we used the identity $H(A, B) = H(A|B) + H(B)$. We denote $R$ as the coefficient of redundancy, which measures the difference in mutual information between the variables, $I(\boldsymbol{S}) \equiv I(S_1; \dots, S_k)$, and the mutual information of the variables conditioned on $E$, $I(\boldsymbol{S}|E)$. When $I(S_i; S_j) < I(S_i; S_j|R)$, the signals contain less mutual information in the absence of the event (we gain information by considering the event), and $R(S_i; S_j; X) < 0$. In this case agents experience a positive benefit from pooling information, which we call synergy.

To demonstrate this effect to higher orders in goups of signals, we perform a similar calculation for a three-signal interaction.

$$\begin{aligned}\frac{\Delta^3 H(E)}{\Delta S_i \Delta S_j \Delta S_k} =& H(E) - H(E|\{S_i, S_j, S_k\}) \\ =& H(S_i) + H(S_j) + H(S_k) - H(S_i|E) - H(S_j|E) - H(S_k|E) - H(S_i, S_j) - H(S_j, S_k) \\ & - H(S_k, S_i) + H(S_i, S_j|E) + H(S_j, S_k|E) + H(S_k, S_i|E) + H(S_i, S_j, S_k) - H(S_i, S_j, S_k|E) \\ =& H(S_i, S_j, S_k) - H(S_i|S_j) - H(S_j|S_k) - H(S_k|S_i) + H(S_i|S_j, E) + H(S_j|S_k, E) \\ & + H(S_k|S_i, E) - H(S_i, S_j, S_k|E) \\ =& I(S_i; S_j; S_k) - I(S_i; S_j; S_k|E) \equiv R(E; S_i, S_j, S_k).\end{aligned} \tag{4}$$

We see that an analogous redundancy coefficient arises in three dimensions. This can generally be retrieved for arbitrary number of dimensions through a similar iterative procedure. We refer to the sum of these moments collectively as the redundancy of the joint distribution, denoted $R_P$ [2],

$$\begin{aligned} R_P \equiv & -\sum_{i>j=1}^{l} \frac{\Delta^2 H(E)}{\Delta S_i \Delta S_j} - \cdots - \frac{\Delta^l H(E)}{\Delta S_1 \ldots \Delta S_l} \\ = & \sum_{i>j=0}^{l} \big[ I(S_i;S_j) - I(S_i;S_j|E) \big] + \cdots + I(S_1;\ldots;S_l) - I(S_1,\ldots,S_l|E) \end{aligned} . \tag{5}$$

Note that redundancies of lower order than cardinality of the signal space must be computed over every combination of signals. For example, when $l = 3$, there are three second order redundancy terms.

This expansion generally defines the benefits to cooperation over increasingly higher orders of cooperation (number of signals). This expression can be used to compute the relative strengths of the various orders of interaction for any set of signals and environmental variables, given their conditional distributions.

### B. Kelly Growth rate

Consider an environment with events conditionally dependent on signals characterized by a joint distribution $P(E, \boldsymbol{S})$ for event $E$ and $l$ signals $S$. Consider a cooperative Kelly investment scheme whereby each participant, agent $i$, witnesses signal $s_i \in S_i$, and informs the collective how to invest their shared resources $r$. The mechanics of pooling resources and collectively investing will be discussed below. Kelly's formalism can be adapted by expanding the environmental probability to contain $l$ signals, $P(E, S) \to P(E, \boldsymbol{S})$, as can the betting matrix $X(E|S) \to X(E|\boldsymbol{S})$, where $\boldsymbol{S} = \{S_1, \ldots, S_l\}$. When odds are fair, the Kelly growth rate is given by the returns to each investment, averaged over the probability of that signal, event pair

$$G = \sum_{e,\boldsymbol{s}}^{E,\boldsymbol{S}} p(e,\boldsymbol{s}) \log \frac{x(e|\boldsymbol{s})}{p(e)} . \tag{6}$$

We expand this equation by inserting $p(e, \boldsymbol{s})$ into the numerator and denominator of the log

$$G = \sum_{e,\boldsymbol{s}}^{E,\boldsymbol{s}} p(e,\boldsymbol{s}) \left[ \log \frac{p(e,\boldsymbol{s})}{p(\boldsymbol{s})p(e)} - \log \frac{p(e|\boldsymbol{s})}{x(e|\boldsymbol{s})} \right] . \tag{7}$$

These two terms can be simply expressed as $G = I(E;\boldsymbol{S}) - \mathrm{E}_{\boldsymbol{s}}\big[D_{KL}\big(P(E|\boldsymbol{s})||X(E|\boldsymbol{s})\big)\big]$, similar to previous work, but we can decomposes this equation in terms of redundant information across the signals using equations (2) and (5).

### C. Information for UXOR circuits

Here we compute $I(E;\boldsymbol{S})$ for the UXOR logic circuit. This represents the information that a group of agents with $l$ distinct signals have about the output $E$ of the probabilistic gate, averaged over all configurations of the gate for a uniformly distributed prior. Because the signals $s_i$ are independent Bernoulli trials with probability 1/2,

$$I(E;\boldsymbol{S}) = \sum_{e,\boldsymbol{s}} P(e,\boldsymbol{s}) \log \frac{P(e,\boldsymbol{s})}{P(e)P(\boldsymbol{s})} = \frac{1}{2^l} \sum_{e,\boldsymbol{s}} P(e|\boldsymbol{s}) \log \frac{P(e|\boldsymbol{s})}{P(e)} .$$

Then, using the fact that the output $E$ is also a Bernoulli variable, $P(e = 1|\boldsymbol{s}) = 1 - P(e = 0|\boldsymbol{s})$, and

$$I_l = I(E;\boldsymbol{S}) = \frac{1}{2^l} \sum_{\boldsymbol{s}} g(P(e=0|\boldsymbol{s})) - g(P(e=0)) , \tag{8}$$

where $g$ is a function representing application of the UXOR gate and is defined over binomial parameters $x$ as

$$g(x) = x \log x - (1-x) \log(1-x) .$$

The number of terms in (8) grows exponentially with $l$ and quickly becomes large. When it is sufficiently large, the sum can be approximated by an average. In particular, for uniformly distributed $P(e=0|\boldsymbol{s})$,

$$\frac{1}{2^l}\sum_{\boldsymbol{s}} g(P(e=0|\boldsymbol{s})) \approx \langle g(x)\rangle_{x\sim U(0,1)}.$$

This expectation can be analytically evaluated. With this, (8) gives

$$I_l = \langle g(x)\rangle_{x\sim U(0,1)} - g(1/2) = \log 2 - \frac{1}{2}\,.$$

### D. Mutual Information for incomplete signal sets

Here, we demonstrate that the collective's information about the gate output scales exponentially in the number of cooperants, $k$, as is depicted in Figure 2. Following the previous section, the introduction of the function $g(x) = x\log x + (1-x)\log(1-x)$ simplifies the expression for mutual information

$$I_k = I(E;\boldsymbol{S}) = \frac{1}{2^k}\sum_{\boldsymbol{s}} g(P(e=0|\boldsymbol{s})) - g(P(e=0))\,. \tag{9}$$

As before, this sum can be interpreted as an average over the uniform distribution when the number of terms is large. Here, the removal of parts of the signal changes the distribution of parameters, so the measure that approximates this sum also changes. We call this new measure $P_k$. Furthermore, whereas in the main text, the subscript of $\boldsymbol{S}_g$ denoted the signals of group $g$, here the subscript of $\boldsymbol{S}_k$ will denote the signal set of cardinality $k$ to be marginalized. With this new notation, the first term in (9) may be written approximately as:

$$\frac{1}{2^k}\sum_{\boldsymbol{s}_k} g(P(e=0|\boldsymbol{s}_k)) \approx \langle g(x)\rangle_{x\sim P_k}.$$

To compute $P_k$ for $k<l$, we need to calculate how the probability of $E$ conditional on the remaining signals $\boldsymbol{s}_k$ changes under the removal of the $k^{\text{th}}$ signal. For this model,

$$P(e|\boldsymbol{s}_{k-1}) = \frac{1}{2}\left(P(e|\boldsymbol{s}_{k-1}, s_k=0) + P(e|\boldsymbol{s}_{k-1}, s_k=1)\right). \tag{10}$$

By iterating this sum, we reduce the number of parameters required to describe $P_k(k)$, which in the main text are given by the set of binomial parameters $\boldsymbol{p}$. Additionally, the distribution $P_k(x)$ becomes increasingly narrow, centered around 1/2, which is the mean of all probabilities $P(e|\boldsymbol{s})$. Parameterizing $P_k(x)$ by its moments allows us to directly compute the mutual information. The moment expansion of the distribution is given by

$$I_k \approx \langle g(x)\rangle_{P_k} - f\left(\frac{1}{2}\right) = \sum_{a=0}^{\infty}\frac{1}{a!}f^{(a)}(x_0)\langle(x-x_0)^a\rangle_{P_k} - f\left(\frac{1}{2}\right).$$

Using standard arguments, which we provide in the following section, these moments approximately scale like

$$m_{k-1}^{(a)} = \left\langle\left(x-\frac{1}{2}\right)^a\right\rangle_{P_{k-1}} \approx \frac{m_k^{(a)}}{2^{a/2}}\,,\qquad m_l^{(a)} = \frac{1}{(a+1)2^a}\,, \tag{11}$$

which is related to the onset of central limit theorem behavior. This provides us with a heuristic explanation for why $I_k$ scales as $1/2^{\lambda}$. After only a few cooperants are removed, higher order terms in the expansion (with order denoted by $a$) quickly die away, leaving only the second-order term

$$I_k \approx \sum_{n=0}^{\infty}\frac{f^{(a)}(1/2)}{a!}m_k^{(a)} \to 2m_k^{(2)} + O((m_k^{(2)})^2)\,.$$

Once this occurs, we can see plainly that between each marginalization the mutual information reduces by half,

$$\frac{I_k}{I_{k+1}} \to \frac{1}{2}.$$

While this explanation gives approximately the correct scaling behavior, it does not admit a good estimate of $I_k$ near full cooperation, since there the higher-order terms in the expansion are not small. To explicitly include these terms, we need all derivatives of $f$, evaluated at $x = 1/2$

$$f^{(a)}(1/2) = \frac{(-1)^a 2^a a!}{a(a-1)}.$$

Inserting these derivatives and the approximation (11) for moments of $P_k$ gives an approximation of $I_k$ as a series. Then, evaluating this series analytically yields a closed form expression.

$$\begin{aligned} I_k &= \sum_{a=0} \frac{f^{(a)}(1/2)}{a!} m_k^{(a)} - g(1/2) \approx \sum_{a=1}^{\infty} \frac{(2a+1)!}{(2a-1)!} \frac{1}{2^{\lambda a}} \\ &= \frac{1}{2} \left[ (2^{-\lambda/2} + 2^{\lambda/2}) \operatorname{arctanh}(2^{-\lambda/2}) + \log(1 - 2^{-\lambda}) - 1 \right], \qquad \lambda > 0 \end{aligned}$$

This expression gives a good approximation for $I_k$ in the small $\lambda$ regime and also captures the scaling in the intermediate regime. For $\lambda \to \infty$, an estimate of the asymptotic behavior is given by setting $z = 2^{-\lambda/2}$ and Taylor expanding around 0.

$$\begin{aligned} \frac{1}{2} \left[ (z + z^{-1}) \operatorname{arctanh}(z) + \log(1 - z^2) - 1 \right] &\to \frac{1}{2} \left[ (z + z^{-1})(z + \frac{1}{3} z^3 + O(z^4)) - z^2 + O(z^4) - 1 \right] \\ &= \frac{1}{6} z^2 + O(z^4) \end{aligned}$$

Because $z = 2^{-\lambda/2}$, the quadratic leading term agrees with the observed exponential scaling $I_k \propto 2^{-\lambda}$. Although the scaling prediction is correct, there is not a regime where this high-$\lambda$ expression consistently estimates $I_k$ in its actual value. The essential reason is that as $k$ decreases, the number of terms in the sum over remaining signal states decreases, and the approximation of that sum as an average over $P_k(x)$ begins to break down. This manifests as an error in the overall scale of the estimate, but not in the exponential dependence on $k$.

Instead, using the fact that $I_l$ is known to a very good approximation and further that information is approximately exponential in $k$, a good estimate for $I_k$ at large to intermediate $k$ is given by

$$I_k = 2^{-\lambda} I_l = 2^{-\lambda} \left( \log 2 - \frac{1}{2} \right).$$

This is the quantity quoted in Figure 7 in the main text.

### 1. *Moment scaling with k*

The following argument justifies (11) and is standard. We produce it here for completeness. Upon moving from $k$ to $k-1$ cooperants, the new conditional distribution is given by (10). This means that $P_{k-1}$ is given by a convolution of $P_k$ with itself.

$$P_{k-1}(x) = \int_{\mathbb{R}^2} dy\, dz\, \delta \left( x - \frac{1}{2}(y+z) \right) P_k(y) P_k(z) = 2 \int_{\mathbb{R}} dy\, P_k(2x - y) P_k(y)$$

The characteristic function of a distribution over a continuous variable is its Fourier transform. Since $P_{k-1}$ is a convolution, its characteristic function is the product of characteristic function of $P_k$ with itself. A slightly more convenient object to work with is therefore the logarithm of the characteristic function:

$$\varphi_k(z) = \log \int_{\mathbb{R}} dx P_k(x) e^{izx}$$

The sum rule above gives a recursion relation for $\varphi_k$

$$\varphi_{k-1}(z) = 2\varphi_k(z/2) + \log 2\,.$$

Now, the cumulants of $P_k$ can be calculated from $\varphi_k$

$$c_k^{(a)} = \frac{1}{i^a} \frac{d^a}{dz^a} \varphi_k(z) \bigg|_{z=0},$$

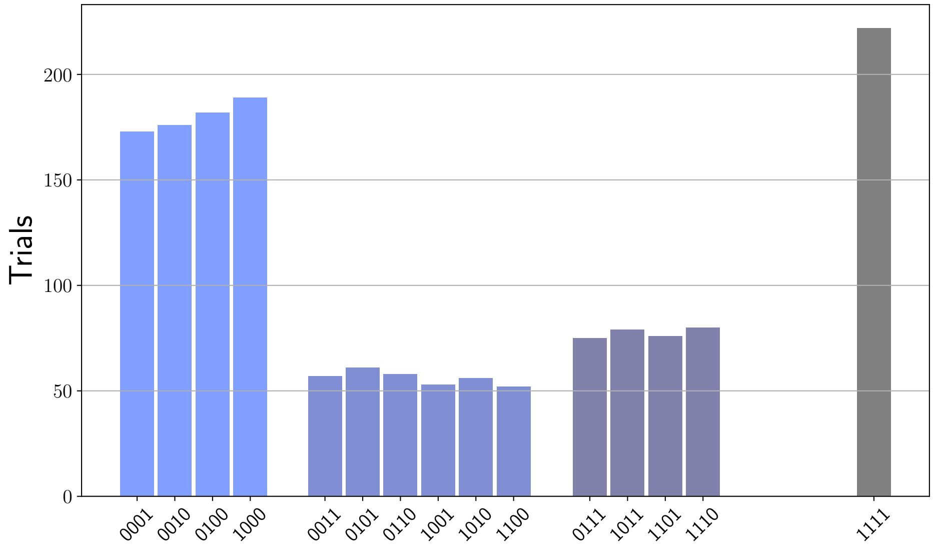


FIG. S1. Trial count by group identification for N=5000. Zeros denote the exclusion of a signal, and ones the inclusion.

Meaning there are also recursion relations for the cumulants:

$$c_{k-1}^{(a)} = 2^{1-a} c_k^{(a)} \tag{12}$$

This leads directly to the central limit theorem, since when the second cumulant is rescaled to remain constant with respect to $n$, all higher-order cumulants scale to zero. Here, by using the fact that the $n^{\text{th}}$ moment $m_k^{(a)}$ can be expressed in terms of cumulants $c_k^{(b)}$ for $b \leq a$, we can see that the second-order cumulant dominates all of these expressions once $k$ is sufficiently small. For example, the fourth moment quickly scales like $(m^{(2)})^2$ as $k$ is decreased from $l$ because the second cumulant begins much larger than the fourth cumulant and remains dominant.

$$\begin{aligned} c_k^{(2)} &= 2^{-\lambda} c_l^{(2)} = \frac{1}{12 \cdot 2^{\lambda}} \\ c_k^{(4)} &= 2^{-3\lambda} c_l^{(4)} = \frac{-1}{120 \cdot 2^{3\lambda}} \\ m_k^{(4)} &= c_k^{(4)} + 3(c_k^{(2)})^2 \approx 3(c_k^{(2)})^2 \sim 2^{-2\lambda} \end{aligned}$$

Hence, due to (12), $m_{k-1}^{(4)} \approx m_k^{(4)}/4$, which agrees with (11).

### E. Monte-Carlo Simulation Details

To study the dynamics of resources in the UXOR environment, we simulated agent investments in a Monte Carlo sampled environment. We randomly assigned $N = 5000$ agents signals in an $l = 4$ environment, then randomly assigned them to groups sized $l \leq N_g \leq 11$. This results in an ensemble of groups with cooperants $1 \leq k_g \leq 4$. Each instance of a group represents one sample of that group configuration. This random assignment scheme, selected for convenience, results in a different sample size for each group identity, where identity is given by the types of signals present. The sample size of a group type scales inversely with the number of combinations of signals of each order $l$. However, each group combination is sampled between 50 and 225 times, resulting in resolvable statistical behavior across all group types. Figure S1 gives the statistics of the groups simulated in Figure 4 of the main text.

The entire population, divided into groups, is instantiated in an environment with the same environmental parameters. The UXOR gate is defined with parameters: $p_1 = .1473$, $p_2 = .7236$, $p_3 = .46451306$, $p_4 = .7068$, $p_5 = .4658$, $p_6 = .3594$, $p_7 = .9708$, $p_8 = .3709$, $p_9 = .8788$, $p_{10} = .7094$, $p_{11} = .7972$, $p_{12} = .6380$, $p_{13} = .4327$, $p_{14} = .2238$, $p_{15} = .4044$, $p_{16} = 0.15949486$. These parameters were discovered from a random search of parameter space and were selected for their convenient qualitative properties.

During each step of the simulation, we reveal Bernoulli-sampled signals to the groups, whose agents make collective decisions on which events to allocate resources. In this sense, every agent in the group views the same signals, and the

group decides the subspace of signals available via the members. Allocations and resource and information rewards are processed identically to prior works, where an LDA defines the update to a group's decision parameters. For each group, we track the resources of a representative agent, informed by the group, investing their individual resources through time.

[1] Bettencourt, L. The rules of information aggregation and emergence of collective intelligent behavior. *Topics In Cognitive Science.* **1**, 598–620 (2009)

[2] Bettencourt, L., Gintautas, V, and Ham, MI. Identification of functional information subgraphs in complex networks. *Physical review letters.* 100, 23, 238701. APS. (2008)
