# Supplementary figures and images for: Information synergy maximizes the growth rate of heterogeneous groups
Source: PNAS Nexus. 2024 Feb 12;3(2):pgae072. doi: 10.1093/pnasnexus/pgae072 (PMC10901557; doi:10.1093/pnasnexus/pgae072)

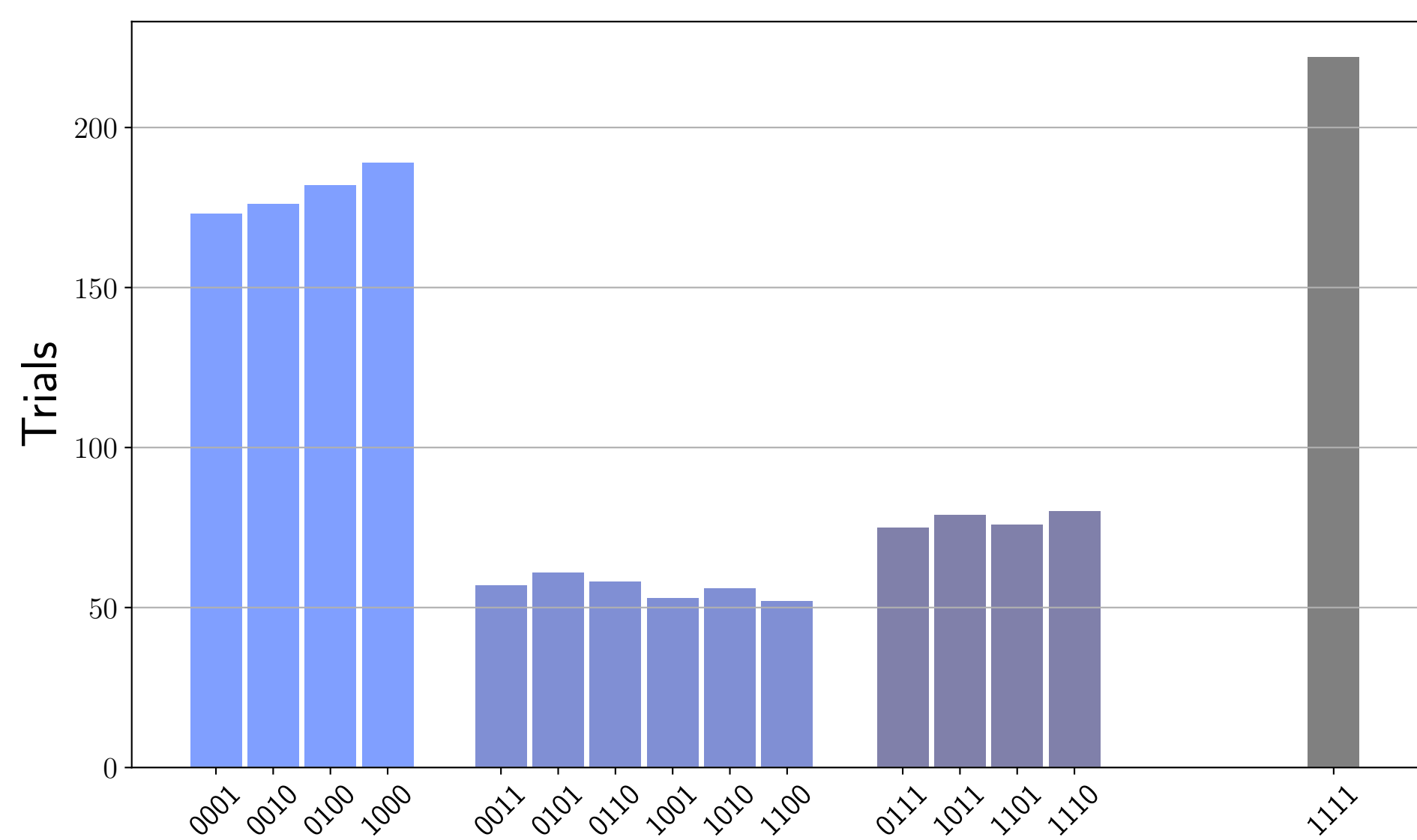

Supplement: pgae072_Supplementary_Data [file pgae072_supplementary_data.zip › PNASNEXUS-PNASNEXUS-2023-00975R-s02.pdf]
